# Development and validation of a simulation-based assessment of operative competence for higher specialist trainees in general surgery

**DOI:** 10.1007/s00464-024-11024-1

**Published:** 2024-07-17

**Authors:** Conor Toale, Marie Morris, Adam Roche, Miroslav Voborsky, Oscar Traynor, Dara Kavanagh

**Affiliations:** 1 Department of Surgical Affairs, Royal College of Surgeons in Ireland, 121 St. Stephen’s Green, Dublin, Ireland; 2 SIM Centre for Simulation Education and Research, Royal College of Surgeons in Ireland, 123 St. Stephen’s Green, Dublin, Ireland

**Keywords:** Surgery, Surgical education and training, Assessment, Simulation

## Abstract

**Background:**

Simulation is increasingly being explored as an assessment modality. This study sought to develop and collate validity evidence for a novel simulation-based assessment of operative competence. We describe the approach to assessment design, development, pilot testing, and validity investigation.

**Methods:**

Eight procedural stations were generated using both virtual reality and bio-hybrid models. Content was identified from a previously conducted Delphi consensus study of trainers. Trainee performance was scored using an equally weighted Objective Structured Assessment of Technical Skills (OSATS) tool and a modified Procedure-Based Assessment (PBA) tool. Validity evidence was analyzed in accordance with Messick’s validity framework. Both ‘junior’ (ST2–ST4) and ‘senior’ trainees (ST 5–ST8) were included to allow for comparative analysis.

**Results:**

Thirteen trainees were assessed by ten assessors across eight stations. Inter-station reliability was high (*α* = 0.81), and inter-rater reliability was acceptable (inter-class correlation coefficient 0.77). A significant difference in mean station score was observed between junior and senior trainees (44.82 vs 58.18, *p* = .004), while overall mean scores were moderately correlated with increasing training year (rs = .74, *p* = .004, Kendall’s tau-b .57, *p* = 0.009). A pass-fail score generated using borderline regression methodology resulted in all ‘senior’ trainees passing and 4/6 of junior trainees failing the assessment.

**Conclusion:**

This study reports validity evidence for a novel simulation-based assessment, designed to assess the operative competence of higher specialist trainees in general surgery.

**Graphical abstract:**

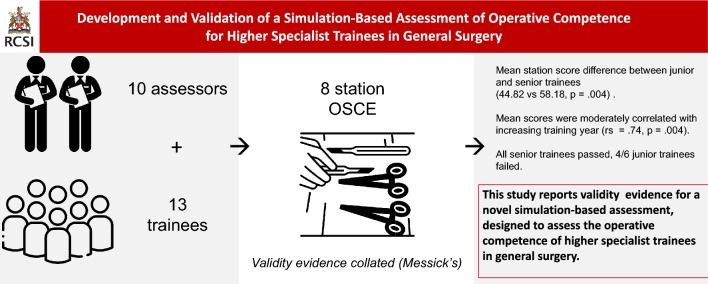

**Supplementary Information:**

The online version contains supplementary material available at 10.1007/s00464-024-11024-1.

## Introduction

The traditional Halstedian apprenticeship training model in surgery has undergone significant revolution in recent decades [1]. Evolving patient expectations regarding the role of surgical trainees in their care [2], an increased emphasis on theater efficiency [3] and well-explored concerns regarding the perceived operative competence and confidence of graduating trainees [4–6], has led to a re-evaluation of this training paradigm. ‘Competency-based’ approaches to outcome-driven training and assessment are well established across training jurisdictions [7, 8], leading to the development of nominally time-independent postgraduate surgical programs [9]. The surgical specialties pose a unique challenge in this regard, due to the requirement for robust and reliable methods of teaching and assessing competence in operative skill [10].

From August of 2021, surgical training in Ireland and the United Kingdom (UK) has become explicitly outcomes based [11]. Trainees are measured against high-level outcomes, (i.e., ‘Capabilities in Practice’). Some trainees who demonstrate accelerated development may complete training more rapidly than the indicative time [11]. A somewhat similar system of high-level ‘milestones’ and derived ‘Entrustable Professional Activity’ assessments now operates in the USA context [8]. In order to transition to a truly competency-based, time-independent training paradigm, operative skill will need to be assessed using objective, standardized, and validated approaches. The primary tool for measuring operative competence in the UK and Ireland context remains the Procedure-Based Assessment, a workplace-based procedure-specific assessment tool [12]. Though this tool has accrued substantial validity evidence, concerns exist regarding the opportunistic way in which these assessments are undertaken by trainees, as well as their potential for rater subjectivity [13]. Operative experience targets, though a traditional proxy measure of operative competence, have been de-emphasized in the updated curriculum and lack validity evidence in the UK and Irish context [13].

The role of simulation-based training and assessment in surgery has evolved in tandem with competency-based education [14]. Simulation is commonplace in surgical training curricula [15] and is increasingly being deployed as an assessment modality [16, 17]. Simulation has most notably been used in high-stake assessments through the Colorectal Objective Structured Assessment of Technical Skill (COSATS) by the American Board of Colon and Rectal Surgery [16, 18]. Such simulation-based assessments have not yet been used in a high-stakes setting for higher specialist training in Ireland or the UK to date. We hypothesize that simulation could be used as a reliable, valid, objective, and standardizable method of summative operative competence assessment for trainees undertaking higher specialist training in general surgery.

This study sought to develop and validate a pilot simulation-based assessment of operative competence based on the common training curriculum of the UK and Ireland; the Joint Committee on Surgical Training (JCST)/Intercollegiate Surgical Curriculum Program (ISCP) curriculum in general surgery. Herein, we describe the approach to assessment design, development, and pilot testing and report on the validity of this assessment in line with Messick’s validity framework [19].

## Methods

### Initial development of the examination framework

The approach to assessment development and validation was derived from a primer for the validation of simulation-based educational assessment published by Cook and Hatala [20]. The primary interpretation of the intended construct was defined as “learners have operative skill sufficient to operate safely as an independent practitioner in General Surgery.” With reference to the UK and Ireland surgery curriculum, this construct relates to the following Capabilities in Practice (CiPs), Generic Professional Capabilities (GPCs), and syllabus requirements: ‘manages an operating list’ (CiP), ‘professional skills’ (GpC), and both ‘technical skills—general’ and ‘technical skills—index procedures’ (syllabus). The intended decision related to this examination was derived through an iterative process of literature review [21], semi-structured interviews with key stakeholder representatives [22], and a survey of surgical trainees regarding their perceptions and experience of simulation-based assessment (unpublished data). This pilot assessment was derived to explore whether scores achieved could inform, as part of a multi-faceted assessment framework, whether a given trainee has sufficient procedural competence to proceed to the next stage of training or independent practice.

This assessment was designed based on the well-established Objective Structured Assessment of Technical Skill (OSATS) examination [23] as followed by other high-stakes simulation-based assessments [16, 18]. Therefore, the initial design of this assessment was that of a time-limited eight-station framework [23]. Operative procedures for use in this assessment were selected using a modified Copenhagen Academy for Medical Education and Simulation Needs Assessment Framework (CAMESNAF) [24]. This resulted in a prioritized list of procedures suitable for simulation-based assessment of general surgery trainees at the end of phase 2 (ST6), from which eight were chosen for inclusion in the assessment model [25]. Future, varying assessment iterations could be created using the procedural lists generated from this Delphi process.

### Assessment instruments

Performance was assessed at each station using a modified Procedure-Based Assessment (PBA) tool (consisting of a task-specific checklist and a global rating scale) [26] and the OSATS tool [27]. The OSATS tool is the most commonly used tool in surgical assessment research [28]. The PBA tool consists of a procedure-specific checklist containing pre-, intra- and post-operative domains, along with a global rater from 1a to 4b. A combination of an equally weighted checklist tool and OSATS tool is currently used by RCSI’s ‘Operative Surgical Skill’ assessments which inform decisions on progression from Core to Higher specialist training [29]. These annual assessments undergo rigorous annual standard setting and validity testing, although focus on core basic surgical skills and tasks rather than procedures. Due to time and feasibility constraints, key component tasks, rather than entire procedures, were simulated for assessment in some stations in this assessment. For this reason, the intra-operative domain of the PBA tool was adapted by a steering group of simulation and surgical education researchers (CT, MM, DOK). Items on the modified checklist were scored as ‘done,’ ‘partially done,’ or ‘not done,’ with total scores standardized and weighted equally to OSATs scores to calculate the total final score. An example assessment tool is available in the supplemental material (Fig. S1, supplemental data). The modified PBA checklists were trialed by a consultant surgeon and surgical trainee to ensure that they could be completed on the proposed stations in the indicated time frame. Data from this trial were not included in subsequent analysis. As assessment tool modifications can have impacts on their validity, this study also seeks to generate validity data for these modified tools.

The LapSim™ simulator was used to assess performance in three of the eight stations. This simulator reports a number of computer-measured metrics (Table S1, supplementary data). LapSim™ recorded metrics did not contribute to candidate scores, although statistical analyses are reported herein.

### Participants

Previous literature has suggested that 8 stations provide a reliable indicator of performance in a multi-station OSCE [23]. While this pilot assessment may inform future sample size considerations in larger iterations, initial sample size was determined based on the number of participants required to demonstrate a difference in outcomes between ‘senior’ and ‘junior’ general surgery trainees (trainees in their last or first four years of surgical training, respectively). Studies reporting mean PBA scores awarded with standard deviations are lacking since the introduction of a revised global rating scale in 2017 [13]. Furthermore, a novel, modified PBA tool for use in simulation-based assessment is being used in this study. For these reason, power calculations could only be conducted using the OSATS tool. Sample size was determined based on anticipated differences in overall scores across the eight-station OSCE format and not at the individual station level. Mean OSATS scores and standard deviations were derived from a prior study using a simulation-based assessment to assess surgeons at the end of training (mean score 0.75, SD 0.06) [30]. Assuming that a score difference of 20% or more was a relevant difference between groups, a sample size of six participants per assessment group was sufficient to minimize the risk of a type II error (*α* = 0.05). Again, it is important to emphasize the pilot nature of this assessment, and the lack of data reporting mean scores across simulated PBA assessment tools let alone the modified checklists used in this study.

### Procedure

Ethical approval was granted by the University of Medicine and Health Sciences at the Royal College of Surgeons in Ireland. The assessment took place at the National Surgical Training Center, Royal College of Surgeons in Ireland, which contains a purpose-built ‘wet lab’ for simulation-based technical skills training and assessment. Examiners were familiarized with the format, tools, and models prior to the assessment, and pre-assessment standard setting was conducted using a modified Angoff method [31]. Candidates were informed of the assessment format in a 30-min briefing session prior to commencement. This orientation session also allowed for introduction to the virtual reality models used (LapSim, Surgical Science, Sweden). Examiners were not informed of the trainee’s level of training. Stations utili**z**ed a combination of bio-hybrid models (ileostomy reversal, operative management of a fistula-in-ano, pilonidal sinus excision, ventral hernia repair, emergency laparotomy, and management of a blunt liver injury) and virtual reality models (laparoscopic appendicectomy, laparoscopic cholecystectomy, and right hemi-colectomy—vessel ligation). The LapSim simulator (Surgical Science, Sweden) was chosen for use in the virtual reality stations given the substantial validity evidence published with respect to its use in training and assessment [32]. Candidates rotated through the stations and were assessed by a single assessor using the modified PBA and OSATs tools. Two stations (ileostomy reversal and emergency laparotomy) were also scored by a second, independent rater. Performance in each station was video recorded in an anonymized fashion for quality assurance purposes. Scores were entered immediately to a secure database (QPERCOM). Instructions regarding the procedure or task to be completed were given to participants before each station. Video capture was used for quality control purposes. Rater training was conducted before the assessment. All assessors were registered consultant surgeon trainers with the Royal College of Surgeons in Ireland.

### Statistical analysis

Validity evidence was collated according to Messick’s validity framework [19]. Internal consistency was assessed using Cronbach’s alpha. Inter-rater reliability was calculated using intra-class correlation coefficients. Reliability was further explored using generali**z**ability theory [33]; variance component analysis was used to determine the relative contribution of trainees, assessors, and stations to observed score variance allowing for the estimation of the ‘true’ or ‘intended’ variance, i.e., the proportion of score variance attributable to individual trainees. For the purpose of this analysis, trainees, assessors, and stations were thought of as a sample from an infinite universe of potential trainees, assessors, and stations [34]. The Consequential validity evidence was explored by generating a pass-fail cut-off score, using borderline regression methodology [35]. A linear regression model was used to plot the global competency score against both OSATS and PBA checklist scores. The global score representing the ‘borderline’ candidate was inserted into the linear equation, and the corresponding total OSATS and PBA checklist scores were extrapolated, representing the true pass score for each station. The pass score for the assessment as a whole was the sum of the pass scores for each station. OSATS and modified PBA scores were equally weighted. Relationships with other variables (training year) were performed using Pearson correlation coefficients. Findings are reported according to the domains of Messick’s unified validity framework; response process and content domains are outlined above, and no further supporting evidence according to these domains is further outlined.

## Results

Thirteen candidates undertook the assessment across eight 15-min stations. Candidates were divided into ‘junior’ trainees in their first 4 years of surgical training (ST1–ST4, *N* = 6) and ‘Senior’ Trainees in their last 4 years of training (ST5–ST8, *N* = 7). Mean scores awarded are outlined in Table 1.

**Table 1:** Mean scores awarded across assessment stations

Station	Mean standardized PBA score (SD)	Mean OSATS (SD)	Mean Total Score (SD)	Mean PBA Global Rater (SD)	Mean competency rater (SD)
1	26.65 (± 6.15)	25.08 (± 4.39)	51.73 (± 10.29)	5.46 (± 0.88)	1.62 (± 0.65)
2	30.24 (± 6.74)	30.76 (± 5.96)	61.01 (± 12.41)	6 (± 1.58)	1.38 (± 0.65)
3	29.72 (± 7.68)	25.07 (± 4.65)	54.79 (± 11.35)	5.84 (± 1.68)	1.77 (± 0.44)
4	13.83 (± 11.59)	23.54 (± 7.86)	37.37 (14.64)	5.69 (± 1.75)	1.00 (± 1.00)
5	26.56 (± 7.33)	26.17 (± 3.07)	52.73 (± 10.25)	5.42 (± 1.24)	1.42 (± 0.90)
6	27.12 (± 12.85)	30.69 (± 4.21)	57.82 (± 15.21)	5.62 (± 1.66)	1.69 (± 0.63)
7	17.5 (± 8.36)	21.31 (± 3.95)	38.81 (± 12.21)	3.54 (± 2.29)	0.46 (± 0.78)
8	31.41 (± 5.52)	32 (± 4.51)	63.41 (± 9.02)	6.83 (± 1.80_	1.92 (± 0.29)

Station 1—fistula-in-ano, 2—ileostomy closure, 3—right hemi-colectomy (vessel ligation), 4—pilonidal sinus excision, 5—ventral hernia repair, 6—laparoscopic appendicectomy, 7—emergency laparotomy (blunt liver trauma), 8—laparoscopic cholecystectomy. SD indicates standard deviation. A competency rater score of 0 indicates a ‘fail/ not competent’ score, 1 indicates ‘borderline,’ and 2 indicates a ‘pass/competent’ score. PBA Global rater scores range from 0 to 8 (see supplemental data)

### Internal structure

Inter-station reliability for the total score awarded was assessed using Cronbach’s alpha (α), calculated as 0.81 for the assessment overall. Removing station 1(management of a fistula-in-ano) would result in a higher α (Table 2). Internal consistency of individual assessment tools is outlined in Table S2, supplementary data. Correlations between individual station scores and the total score and between scores awarded at each station, are reported in Tables S3 and S4 (supplementary data).

**Table 2:** Assessment metrics for each station

Station	Cronbach’s alpha if item deleted	*R*^2^	Inter-grade discrimination	Number of failures
1	.825	.76	11.95	4
2	.787	.78	14.83	3
3	.786	.81	20.88	2
4	.790	.47	6.88	9
5	.804	.93	10.55	4
6	.744	.45	10.87	3
7	.743	.86	13.52	9
8	.799	.68	21.3	0

Total number of candidates (*N*) = 13

Inter-rater reliability was assessed for two stations: emergency laparotomy (Station 7) and ileostomy reversal (Station 2). On intra-class correlation coefficient analysis, inter-rater reliability was 0.77. Individual station scores variably correlated with the total score awarded. Using Generalizability Theory, a reliability coefficient (G) of 0.7 was calculated for the overall 8-station assessment. Fourteen hypothetical stations would be required to increase the reliability coefficient to > 0.8. Further, data relating to the model construction and variance component analysis are outlined in Table 3.

**Table 3:** Generalizability theory analysis

Variance component	Variance estimate, total score	Proportion of observed variance (%)
Trainee (Vt)	92.116	38.244
Station (Vs)	54.401	22.586
Trainee × station (Vts)	94.345	39.169
Residual error (Ve)	0.000	0.000
Number of stations required to achieve G ≥ 0.8	6	
Number of stations required to achieve Phi ≥ 0.8	14	

Reliability coefficient ‘G’ is calculated by assessing the intended or true variance, i.e., variance attributable to participants (trainees). This is expressed as (Vt/ (Vt + (Vs/Ns) + (Vts/Ns) + Ve), where Ns is the number of stations. G can be mathematically modeled with increasing numbers of hypothetical stations in order to determine the number of stations required to achieve reliability G ≥ 0.7 or ≥ , conventionally required for low- and high-stakes assessments, respectively

### Relationships with other variables—differentiating between junior and more senior trainees

**Fig. 1:**
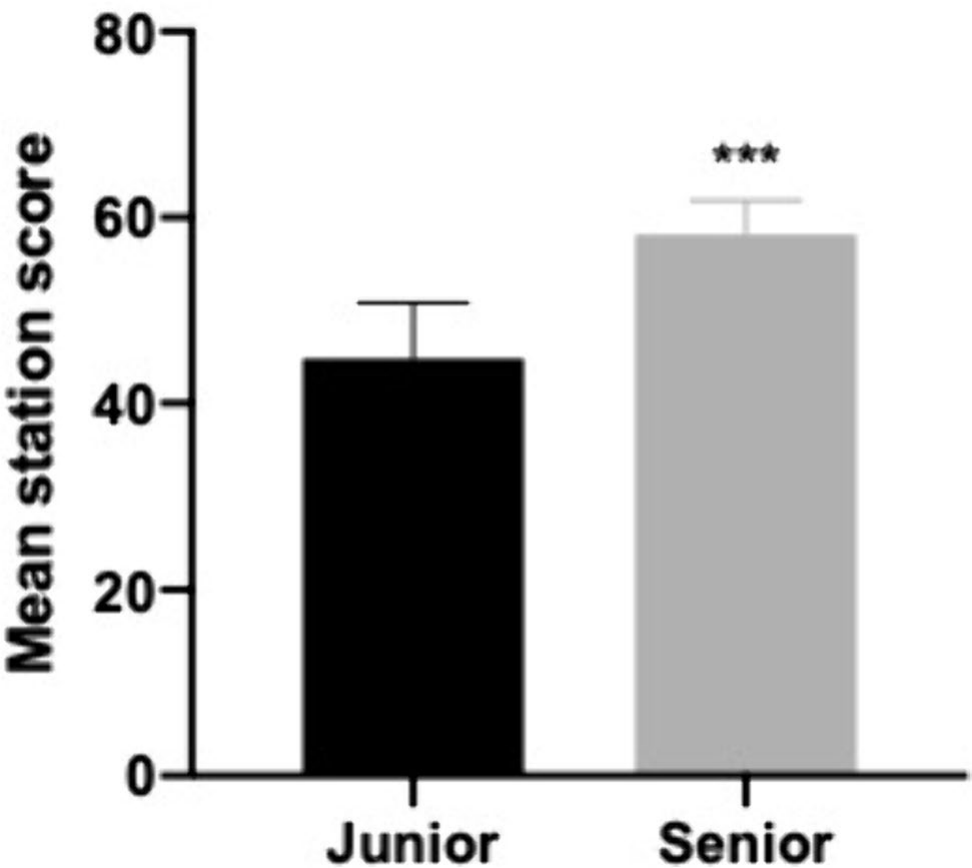
Difference in mean scores achieved by ‘Junior’ (ST2–ST4) trainees and ‘Senior’ (ST5–ST8) trainees. ***Indicates *p* < 0.005

**Fig. 2:**
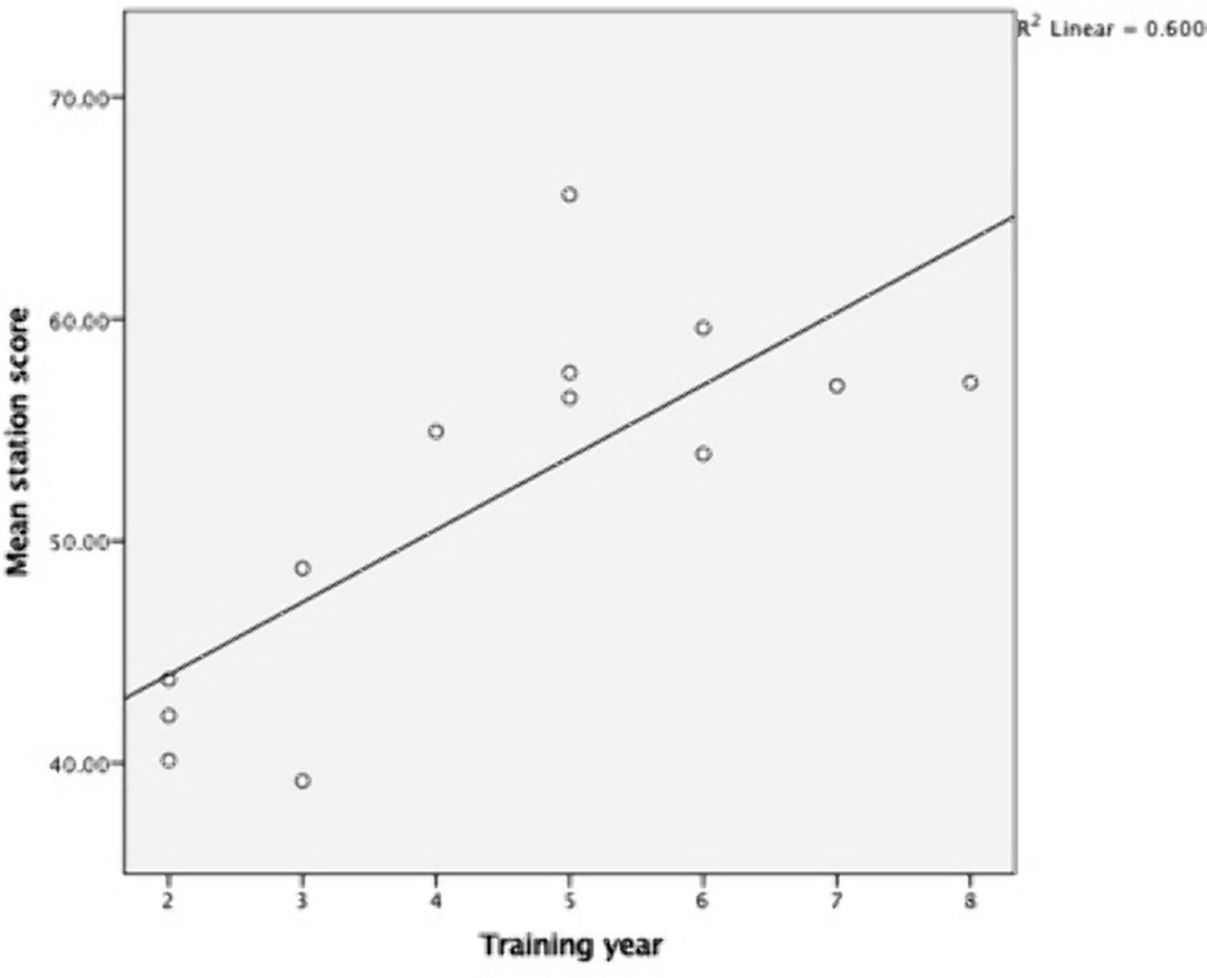
Relationship between training year and mean score obtained

A significant difference in mean station score was observed between ‘Junior’ and ‘Senior’ trainees (44.82 vs 58.18, *p* = 0.004) (Fig. 1). Mean scores were moderately correlated with increasing training year (rs = 0.74, *p* = 0.004, Kendall’s tau-b 0.57, *p* = 0.009) (Fig. 2).

At an individual station level, five of eight stations could differentiate between ‘Junior’ and ‘Senior’ trainee candidates (Table 4).

**Table 4:** Mean score differences for ‘Junior’ vs. ‘Senior’ trainees across individual stations

Station	Group	Mean	Std. deviation	Sig. (2-tailed)
1. Fistula-in-ano	Junior	45.47	9.12	**.04**
	Senior	57.10	8.35	
2. Ileostomy closure	Junior	52.69	14.47	**.02**
	Senior	68.14	2.25	
3. Right hemi-colectomy	Junior	47.40	12.22	**.022**
	Senior	61.13	5.64	
4. Pilonidal sinus excision	Junior	32.05	10.95	.24
	Senior	41.92	16.63	
5. Ventral hernia repair	Junior	49.92	10.40	.37
	Senior	55.54	10.20	
6. Laparoscopic appendicectomy	Junior	46.46	15.99	** < .01**
	Senior	67.57	3.12	
7. Emergency laparotomy (blunt liver trauma)	Junior	28.21	4.13	** < .001**
	Senior	47.89	8.70	
8. Laparoscopic cholecystectomy	Junior	59.75	10.97	.25
	Senior	66.02	7.06	

Statistically significant results are highlighted in bold (*p* < 0.05).

A number of simulator-measured metrics were further recorded for the three laparoscopic skills (LapSim™) stations (Table S1, supplementary data). Correlations between simulator- and assessor-recorded metrics are outlined in Tables S5, S6, and S7 (supplementary data).

### Consequences

A pass-fail standard for each station was generated using borderline regression methodology. Passing scores and passing rates for individual stations are outlined in Table 5. There was no difference in the pass rate (9/13) when using a compensatory model (where the candidate can fail an unlimited number of stations once the overall pass mark is achieved) or a conjunctive model (where the candidate is required to achieve the overall pass mark and pass at least 50% of stations). Either method resulted in all ‘senior’ trainees passing and 2/6 of ‘junior’ trainees passing. Only one trainee passed all eight stations.

**Table 5:** Borderline regression passing scores and passing rates

Station	Mean score (SD)	Passing score	Passing rate (%)
1. Fistula-in-ano	51.73 (± 10.29)	44.38	69.23
2. Ileostomy closure	61.01 (± 12.41)	55.31	76.92
3. Right hemi-colectomy	54.79 (± 11.35)	38.73	84.62
4. Pilonidal sinus excision	37.37 (± 14.64)	37.36	30.77
5. Ventral hernia repair	52.73 (± 10.25)	48.33	66.67
6. Laparoscopic appendicectomy	57.82 (± 15.21)	50.30	76.92
7. Emergency laparotomy	38.81 (± 12.21)	46.09	30.77
8. Laparoscopic cholecystectomy	63.41 (± 9.02)	43.88	100
Total score	52.01 (± 8.36)	44.82	69.23

*SD* indicates standard deviation

### Feasibility

Two rounds of assessment were conducted. Each session was conducted over 2 h. Models and required equipment for open stations cost an estimated €4680, although much of the reusable equipment was already available within the simulation laboratory at RCSI. Rental and support costs for the LapSim laparoscopic simulators were €4305. Staffing costs were estimated at an additional €4000, bringing the estimated cost of the assessment to €12,985.

## Discussion

This study outlines validity evidence for a novel pilot simulation-based assessment of operative competence, designed for assessment of higher specialist trainees in General surgery and centered around operative competency expectations derived from the intercollegiate surgical curriculum program of Ireland and the UK. The principles of assessment design and validity testing are not specific to this jurisdictional context and can be used to generate simulation-based assessments within competency-based training curricula internationally. High internal consistency metrics were generated, with acceptable inter-rater reliability. While a sufficient number of observations were observed to reliably rank candidates (G > 0.8 with 6 stations), 14 stations would be required in order to reliably determine whether a candidate met the desired level of operative competence across procedures, due to the observed absolute error variance [34]. The assessment can differentiate between trainees according to level of training, with more senior trainees outperforming their junior trainee counterparts across management of a fistula in-ano, ileostomy closure, right hemi-colectomy, laparoscopic appendicectomy, and emergency laparotomy stations. Mean station scores were moderately and significantly correlated with increasing training year (*R*^2^ = 0.6). Using borderline regression methodology, a pass-fail mark was generated which resulted in all trainees in their last 4 years of training passing the assessment and 4/6 more junior trainees failing. The estimated cost of delivering this pilot assessment was €12,985.

Only one participant passed all eight stations. Given the absolute curricular (and indeed, clinical) requirements for competence across several key index procedures, it is arguable that an alternate approach to standard setting and pass/fail cut-off is required. In particular, it could be argued that candidates should be required to pass all stations in order to pass the entire assessment, without the capacity for inter-station score compensation. The ‘patient safety’ method of standard setting [36, 37] is an approach to developing defensible minimum passing standards whereby incorrect performance in critical items or procedures leads to failure without recourse to compensation.

The implementation of competency-based education principles across surgical training programs requires robust methods of trainee assessments. The newly updated general surgery curriculum of the UK and Ireland emphasizes two critical stages during higher specialist training with corresponding operative competency expectations: phase 2 outlining expectation in core elective and emergency procedures and phase 3 outlining further competency expectations in a given trainee’s sub-specialty of interest. Assessment across these procedures is primarily conducted in the workplace. In other jurisdictions, high-stakes simulation-based assessments have been designed to inform end-of-training certification decisions [16–18, 38]. This study suggests that a similar assessment modality has sufficient validity evidence to support its use in the assessment of trainees in Ireland. However, it is important to emphasize that this study reports pilot data with a limited number of trainee participants and does not yet support the implementation of such a modality in informing high-stakes assessment outcomes. The role, if any, of simulation in informing high-stakes training decisions such as progression or end-of-training certification remains to be fully elucidated, particularly in the context of an existing longitudinal multi-faceted assessment program. Furthermore, this study does not further explore the potential value of this assessment, or similar assessments, in a purely formative context.

In a 2012 Delphi study of members of the Association for Surgical Education, clarifying the role of simulation for the certification of residents and practicing surgeons was identified as a high priority for surgical education research [39]. Simulation-based assessments (SBA) currently play varying, though often significant, roles in the training and certification process across jurisdictions. For example, passing the fundamentals of laparoscopic surgery assessment is a certification requirement in certain specialties in the USA [40–44]. For practicing surgeons, the General Medical Council of the UK use simulated procedures, with cadaveric models, to inform performance assessments to support fitness-to-practice decisions [45]. At end-of-training certification level, studies to date suggest that SBA assesses a different construct to knowledge-based assessments and may therefore add validity to the certification process [18, 46]; a low positive correlation only (*r* = 0.25) was observed between scores awarded to trainees in a simulation-based certification exam and oral examination scores by de Montbrun et al. [18]. Despite published validity evidence for such assessments according to modern concepts of construct validity, the acceptability of such assessments remains a barrier to their widespread implementation. Stakeholders report lack of evidence and financial considerations as factors contributing to lack of SBA implementation [47].

These concerns, particularly with respect to cost, are not unfounded. The estimated costs of the reported assessment approached €13,000. This assessment took place in a well-equipped national training center with purpose-designed facilities and trained personnel. The implementation of such assessments across programs and local contexts will therefore likely vary. Furthermore, studies have yet to determine the appropriate role and weighting of simulation-based assessments in the context of programmatic assessment. Potential drawbacks of SBA when compared to workplace-based assessment include insufficient model fidelity, difficulty in simulating case complexity or variation, and further challenges associated with conducting longitudinal, repeated assessments. Finally, the acceptability of such assessments to key stakeholders in surgical education should be further explored by future studies prior to the implementation of any high-stakes assessment.

## Limitations

This study is limited by its small sample size. In particular, pass rates calculated by borderline regression methodology are limited due to low numbers or ‘borderline’ performance scores, particularly in some individual stations. Initial power calculations were derived using published data on the OSATS tool, with limited published data available on mean PBA scores across procedures. The ultimate scoring tool used an equally weighted OSATs and PBA tool. Derived procedures, assessment tools, and competency expectations may differ across jurisdictions, training stages, and local contexts. For example, it may be inappropriate to assess the competence of breast surgery trainees to consultant-ready standard in colorectal procedures, such as ileostomy reversal or fistula-in-ano surgery. During standard setting examiners were informed to assess candidates to the level of a day-1 general surgery consultant regardless of sub-specialty interest. In future iterations it may be required to further outline competency expectations for each procedure in more detail, and in particular emphasize the need to assess to the minimum standard of competence required for all trainees regardless of intended or declared sub-specialty interest. The somewhat unique highly centralized, nationally delivered and relatively well-resourced simulation-based training curriculum in Ireland may mean that further implementation challenges would be encountered by other jurisdictions. A further potential limitation is the use of a compensatory scoring method over a non-compensatory method. Given that trainees at the end of phase 2 are expected to display competent performance in a series of core general and emergency procedures, it may be more appropriate to design an assessment that ensures a baseline procedure-specific competence in all included procedural stations.

## Conclusion

This study reports validity evidence of a novel simulation-based assessment of operative competence, designed for assessment of Irish higher specialist trainees in General surgery. Further larger-scale validation studies, along with studies exploring the role of such assessments in a longitudinal training and evaluation program, will be required prior to the implementation of SBA in general surgery training.

### Supplementary Information

Below is the link to the electronic supplementary material.

Supplementary file1 (DOCX 64 KB)

## Data Availability

Data is available upon request.
