# Supplementary material for: Development and validation of a simulation-based assessment of operative competence for higher specialist trainees in general surgery
Source: Surg Endosc. 2024 Jul 17;38(9):5086–95. doi: 10.1007/s00464-024-11024-1 (PMC11362445; doi:10.1007/s00464-024-11024-1)
Supplement: Supplementary file 1 — Supplementary file1 (DOCX 64 KB) [file 464_2024_11024_MOESM1_ESM.docx]

**Development and Validation of a Simulation-Based Assessment of Operative Competence for Higher Specialist Trainees in General Surgery**

*C Toale^1^, M Morris^1^, A Roche^2^, M Voborsky^2^, O Traynor^1^, DO Kavanagh^1^*

^1^Department of Surgical Affairs, Royal College of Surgeons in Ireland

^2^SIM Centre for Simulation Education and Research, Royal College of Surgeons in Ireland

**Corresponding author:** Conor Toale, Department of Surgical Affairs, Royal College of Surgeons in Ireland

**ORCID ID:** 0000-0002-4858-4813**, Twitter:** @ToaleConor

**Supplementary Materials - Index**

| **Supplementary Methods** |  |
| --- | --- |
| Table S1: LapSim^TM^ recorded metrics | *Page 2* |
| Figure S1: Sample assessment tool | *Page 3* |
| **Supplementary Results** |  |
| Table S2: Internal consistency of assessment tools, assessed using Cronbach’s alpha | *Page 7* |
| Table S3: Correlation (Pearson) between individual station scores and total scores awarded | *Page 7* |
| Table S4 Internal consistency: correlations between score domains at the individual station level | *Page 7* |
| Table S5: Automated LapSim performance metrics for junior vs senior trainees; difference mean results for laparoscopic cholecystectomy by group, junior vs senior trainees | *Page 8* |
| Table S6: Automated LapSim performance metrics for junior vs senior trainees; difference mean results for laparoscopic appendicectomy by group, junior vs senior trainees | *Page 7* |
| Table S7: Automated LapSim performance metrics for junior vs senior trainees; difference mean results for laparoscopic right hemi-colectomy by group, junior vs senior trainees | *Page 8* |

**Supplementary Methods**

**Table S1**

LapSimTM recorded metrics:

Laparoscopic Cholecystectomy:

- Left instrument path length (m)
- Left instrument angular path (degrees)
- Right instrument path length (m)
- Right instrument angular path (degrees)
- Left instrument outside view (#)
- Left instrument outside view (s)
- Right instrument outside view (#)
- Right instrument outside view (s)
- Blood loss (ml)
- Energy applied in air (s)
- Missing clips (#)
- Fatal clips (#)
- Missing cuts (#)
- Number of cuts on the liver (#)
- Number of cuts on the gallbladder (#)
- Ducts/ vessels ripped (#)
- Ducts/ vessels ablated (#)
- Number of fatal dissections (#)
- Total time burned on arteries (s)
- Total time burned on ducts (s)
- Total time burned on liver (s)
- Number of times burned on arteries (#)
- Number of times burned on ducts (#)
- Number of times burned on liver (#)
- Number of times burned on gallbladder (#)
- Percent of connective tissue ablated
- Percent of adipose tissue ablated
- Percent of connective tissue ripped
- Percent of adipose tissue ripped

Laparoscopic appendicectomy

- Total time
- Left instrument path length (m)
- Left instrument angular path (degrees)
- Right instrument path length (m)
- Right instrument angular path (degrees)
- Left instrument outside view (#)
- Left instrument outside view (s)
- Right instrument outside view (#)
- Right instrument outside view (s)
- Blood loss (ml)
- Energy applied in air (s)
- Total time burned on the appendix (s)
- Number of times burned on the appendix (#)
- Percent of adhesions ablated
- Percent of mesentery ablated
- Percent of adhesions ripped
- Percent of mesentery ripped
- Evacuation from body (performed/ not performed)
- Incorrect stapler used (yes/ no)

Laparoscopic right hemi-colectomy

- Total time
- Left instrument path length (m)
- Left instrument angular path (degrees)
- Left instrument outside view (#)
- Left instrument outside view (s)
- Right instrument path length (m)
- Right instrument angular path (degrees)
- Right instrument outside view (#)
- Right instrument outside view (s)
- Number of vessel injured (#)
- Correct vessel divisions (#)
- Burn time on artery (s)
- Burn time on vein (s)
- Burn time on duodenum (s)
- Number of burns on artery (#)
- Number of burns on vein (#)
- Number of burns on duodenum (#)
- Percentage of artery ablated
- Percentage of vein ablated
- Total time artery overstretched (s)
- Total time vein overstretched (s)
- Number of times artery overstretched
- Number of veins overstretched
- Artery ripped (yes/no)
- Vein ripped (yes/no)
- Number of pressure damages on duodenum (#)
- Gonadal artery burned (yes/ no)
- Burned on ureter (yes/ no)
- Maryland jaw vessel sealer/divider has been used on wrong structures (yes/no)

**Figure S1 – Sample assessment tool**

**Station 2: Ileostomy closure**

| Trainee number | Assessor | Date |
| --- | --- | --- |
|  |  | 29/08/22 |

**Instructions to trainee:**

A 65-year-old female patient underwent a laparoscopic low anterior resection with a diverting loop ileostomy for rectal cancer approximately 12 weeks ago. She has attended today for elective reversal of her ileostomy. She is currently in the operating theatre under general anaesthetic. The surgical site has been painted and draped. Please proceed with ileostomy reversal. Please demonstrate a stapled side-side anastomosis.

**Procedure-Based Assessment (Intra-Operative Domain)**

| Item | Description | Done | Partially done | Not done |
| --- | --- | --- | --- | --- |
| 1 | Follows an agreed, logical sequence or protocol for the procedure |  |  |  |
| 2 | Consistently handles tissue well with minimal damage |  |  |  |
| 3 | Demonstrates a sound technique of knots and sutures/ staples |  |  |  |
| 4 | Uses instruments appropriately and safely |  |  |  |
| 5 | Proceeds at appropriate pace with economy of movement |  |  |  |
| 6 | Anticipates and responds appropriately to variation (e.g.) anatomy |  |  |  |
| 7 | Deals calmly and effectively with unexpected events |  |  |  |
| 8 | Uses assistant(s) to the best advantage at all times |  |  |  |
| 9 | Mobilises the ileostomy down to the level of the peritoneal cavity |  |  |  |
| 10 | Makes a correct assessment of whether resection of the bowel loop is required (e.g. tests both limbs for serosal tears with saline or betadine under pressure) |  |  |  |
| 11 | Resects appropriate portion of small bowel if required. |  |  |  |
| 12 | Makes a safe closure/ anastomosis of the small bowel |  |  |  |
| 13. | Safely reduces anastomosed/ closed small bowel loop into the peritoneal cavity |  |  |  |
| 14. | Closes fascial layer |  |  |  |
| 15. | Performs purse string closure of skin defect/ otherwise closes skin defect appropriately |  |  |  |

**NEXT PAGE**

**OSATS**

| **Respect for Tissue** | | Tick |
| --- | --- | --- |
| 1. | Frequent and unnecessary force on tissue or caused damage by inappropriate use of instruments |  |
| 2. |  |  |
| 3. | Careful handling of tissue but occasionally caused inadvertent damage |  |
| 4. |  |  |
| 5. | Consistently handled tissue appropriately with minimal damage |  |
| **Time and Motion** | | |
| 1. | Many unnecessary moves |  |
| 2. |  |  |
| 3. | Efficient time/ motion but some unnecessary moves |  |
| 4. |  |  |
| 5. | Clear economy of movement and maximum efficiency |  |
| **Instrument Handling** | | |
| 1. | Repeatedly makes tentative or awkward moves with instruments by inappropriate use of instruments |  |
| 2. |  |  |
| 3. | Competent use of instruments but occasionally appeared stiff or awkward |  |
| 4. |  |  |
| 5. | Fluid moves with instruments and no awkwardness |  |
| **Knowledge of instruments** | | |
| 1. | Frequently asked for the wrong instrument or used inappropriate instrument |  |
| 2. |  |  |
| 3. | Knew names of most instruments and used appropriate instruments |  |
| 4. |  |  |
| 5. | Obviously familiar with the instruments and their names |  |
| **Flow of operation** | | |
| 1. | Frequently stopped operating and seemed unsure of next move |  |
| 2. |  |  |
| 3. | Demonstrated some forward planning with reasonable progression of procedure |  |
| 4. |  |  |
| 5. | Obviously planned course of operation with effortless flow from one move to the next |  |
| **Use of assistants** | | |
| 1. | Consistently placed assistants poorly or failed to use assistants |  |
| 2. |  |  |
| 3. | Appropriate use of assistants most of the time |  |
| 4. |  |  |
| 5. | Strategically used assistants to the best advantage at all times |  |
| **Knowledge of specific procedure** | | |
| 1. | Deficient knowledge. Needed specific instruction at most steps |  |
| 2. |  |  |
| 3. | Knew all important steps of operation |  |
| 4. |  |  |
| 5. | Demonstrated familiarity with all aspects of the operation |  |

**NEXT PAGE**

**Procedure-Based Assessment (Global Summary)**

| Level at which completed elements of the PBA were performed on this occasion | | Tick as appropriate |
| --- | --- | --- |
| Level 0 | Insufficient evidence observed to support a summary judgment |  |
| Level 1a | Able to assist with guidance (was not familiar with all steps of the procedure) |  |
| Level 1b | Able to assist without guidance (Knew all steps of procedure and anticipated next move) |  |
| Level 2a | Guidance required for most/ all of the procedure (or part performed) |  |
| Level 3a | Procedure performed with minimal guidance or intervention (needed occasional help) |  |
| Level 3b | Procedure performed competently without guidance of intervention but lacked fluency |  |
| Level 4a | Procedure performed competently without guidance or intervention |  |
| Level 4b | As 4a and was able to anticipate, avoid and/ or deal with common problems/ complications |  |

**Generic Global Summary**

| Not Competent | Borderline | Competent |
| --- | --- | --- |
|  |  |  |

**Supplementary Results**

*Table S2: Internal consistency of assessment tools, assessed using Cronbach’s alpha*

| **Score** | **α** |
| --- | --- |
| PBA | 0.68 |
| OSATS | 0.85 |
| Total Score | 0.81 |
| PBA global rater | 0.81 |
| Competency rater | 0.71 |

*PBA indicates Procedure-Based Assessment tool, OSATS, Objective Structured Assessment of Technical Skill.*

*Table S3: Correlation (Pearson) between individual station scores and total scores awarded*

| **Station** | **1** | **2** | **3** | **4** | **5** | **6** | **7** | **8** |
| --- | --- | --- | --- | --- | --- | --- | --- | --- |
| **r**  **p** | .48  .10 | .66  .02  * | .63  .02  * | .69  .01  * | .54  .07  * | .89  <.001  *** | .84  <.001  *** | .57  .06 |

*r indicates Pearson correlation coefficient, p, p-value, *, p<0.05, **, p<0.005, ***, p<0.0005*

*Table S4 Internal consistency: correlations between score domains at the individual station level*

| Station 1 – fistula-in-ano | | | | |
| --- | --- | --- | --- | --- |
|  | mPBA | OSATS | Total | mPBA GR |
| OSATS | τb = .83  p <0.001 |  |  |  |
| Total | τb = .94  p <.0001 | τb = .90  p <.0001 |  |  |
| mPBA GR | τb = .77  p = .001 | τb = .83  p <.0001 | τb = .79  p = 0.001 |  |
| Competence | τb = .63  p = .010 | τb = .72  p = .003 | τb = .67  p = 0.005 | τb = .59  p = 0.024 |
| Station 2 - ileostomy closure | | | | |
| OSATS | τb = .68  p = 0.003 |  |  |  |
| Total | τb = .87  p <.0001 | τb = .83  p <.0001 |  |  |
| mPBA GR | τb = .61  p = 0.009 | τb = .66  p = 0.005 | τb = .52  p = .022 |  |
| Competence | τb = .51  p = 0.040 | τb = .57  p = 0.021 | τb = .49  p = 0.05 | τb = .68  p = 0.007 |
| Station 3 - right hemi-colectomy (vessel ligation) | | | | |
| OSATS | τb = .73  p = 0.001 |  |  |  |
| Total | τb = .90  p<.0001 | τb = .84  p <.0001 |  |  |
| mPBA GR | τb = .79  p.= .001 | τb = .73  p – 0.001 | τb = .81  p<.0001 |  |
| Competence | τb = .65  p = .010 | τb = .53  p .033 | τb = .62  p = .011 | τb = .62  p = .019 |
| Station 4 - pilonidal sinus excision | | | | |
| OSATS | τb = 0.013  p = .951 |  |  |  |
| Total | τb = .56  p = 0.007 | τb = .46  p = .032 |  |  |
| mPBA GR | τb = -.11  p = .647 | τb = .80  p = .001 | τb = .33  p = .151 |  |
| Competence | τb = -.099  p = 0.68 | τb = .72  p = .003 | τb = .36  p = .131 | τb = .89  p = .001 |
| Station 5 - ventral hernia repair | | | | |
| OSATS | τb = .69  p = .003 |  |  |  |
| Total | τb = .95  p<.0001 | τb = .75  p = .001 |  |  |
| mPBA GR | τb = .73  p = .003 | τb = .69  p = .006 | τb = .77  p = .001 |  |
| Competence | τb = .72  p = .004 | τb = .79  p = .002 | τb =.73  p = .003 | τb = .74  p = .006 |
| Station 6 laparoscopic appendicectomy | | | | |
| OSATS | τb = .58  p = .011 |  |  |  |
| Total | τb = .79  p<.0001 | τb = .82  p<.0001 |  |  |
| mPBA GR | τb = .64  p = .007 | τb = .73  p = .001 | τb = .70  p = .002 |  |
| Competence | τb = .337  p = .186 | τb = .62  p =.011 | τb = .45  p = .065 | τb = .54  p = .032 |
| Station 7 – emergency laparotomy (blunt liver trauma) | | | | |
| OSATS | τb = .88  p<.0001 |  |  |  |
| Total | τb = .97  p<.0001 | τb = .91  p<.0001 |  |  |
| mPBA GR | τb = . 88  p<.0001 | τb = .89  p<.0001 | τb = .89  p<.0001 |  |
| Competence | τb = .74  p = .002 | τb = .74  p = .002 | τb = .74  p = .002 | τb = .77  p = .003 |
| Station 8 - laparoscopic cholecystectomy | | | | |
| OSATS | τb = .56  p = .026 |  |  |  |
| Total | τb = .72  p =.004 | τb = .87  p<.001 |  |  |
| mPBA GR | τb = .74  p = .004 | τb = .53  p =.038 | τb = .58  p = .020 |  |
| Competence | τb = .38  p = .163 | τb = .47  p = .088 | τb = .44  p = .099 | τb = .39  p = .161 |

*mPBA indicates modified Procedure Based Assessment (checklist) score, OSATS, Objective Structured Assessment of Technical Skills, mPBA GR, mPBA Global Rater, Competence, global competence rater, τb, Kendalls tau-b, p, p-value. Significant correlations are highlighted in bold text.*

Table S5: Automated LapSim performance metrics for junior vs senior trainees; difference mean results for laparoscopic cholecystectomy by group, junior vs senior trainees

|  | Levene's Test for Equality of Variances | | t-test for Equality of Means | | | | | | |
| --- | --- | --- | --- | --- | --- | --- | --- | --- | --- |
|  | F | Sig. | t | df | Sig. (2-tailed) | Mean Difference | Std. Error Difference | 95% Confidence Interval of the Difference | |
|  |  |  |  |  |  |  |  | Lower | Upper |
| Total time | .842 | .380 | .885 | 10 | .397 | 80.08112 | 90.52936 | -121.63087 | 281.79310 |
| Left instrument  path length | .121 | .735 | .828 | 10 | .427 | .93245 | 1.12648 | -1.57750 | 3.44241 |
| Left instrument  angular path | .000 | .991 | .315 | 10 | .759 | 109.72449 | 348.52849 | -666.84538 | 886.29436 |
| Right instrument  path length | .544 | .478 | .358 | 10 | .728 | .60225 | 1.68198 | -3.14544 | 4.34994 |
| Right instrument  angular path | 8.162 | .017 | 1.321 | 10 | .216 | 462.48162 | 350.20296 | -317.81921 | 1242.78244 |
| Left instrument outside view (n) | 1.208 | .297 | .808 | 10 | .438 | 3.91429 | 4.84348 | -6.87765 | 14.70622 |
| Left instrument outside view (s) | .389 | .547 | -1.072 | 10 | .309 | -108.84718 | 101.50742 | -335.01979 | 117.32544 |
| Right instrument outside view (n) | 2.702 | .131 | .751 | 10 | .470 | 5.28571 | 7.03971 | -10.39974 | 20.97117 |
| Right instrument outside view (s) | 4.561 | .058 | 1.161 | 10 | .273 | 12.90408 | 11.11436 | -11.86025 | 37.66842 |
| Blood loss | 6.226 | .032 | 4.266 | 10 | .**002** | .79746 | .18693 | .38095 | 1.21397 |
| Energy applied in air | .778 | .398 | .074 | 10 | .942 | .32297 | 4.33932 | -9.34565 | 9.99158 |
| Missing clips | 2.538 | .142 | -.750 | 10 | .470 | -.48571 | .64751 | -1.92845 | .95702 |
| Fatal clips | 10.370 | .009 | 1.208 | 10 | .255 | .20000 | .16562 | -.16901 | .56901 |
| Number of cuts on the liver | 4.000 | .073 | -.833 | 10 | .424 | -.14286 | .17143 | -.52482 | .23911 |
| Number of cuts on the gallbladder | .866 | .374 | -.564 | 10 | .585 | -.25714 | .45571 | -1.27253 | .75825 |
| Total time burned on arteries | 4.000 | .073 | -.833 | 10 | .424 | -.03185 | .03821 | -.11699 | .05330 |
| Total time burned on liver | .013 | .910 | .526 | 10 | .610 | 6.36167 | 12.08684 | -20.56948 | 33.29282 |
| Total time burned on gallbladder | .045 | .836 | 1.741 | 10 | .112 | 5.83055 | 3.34871 | -1.63084 | 13.29193 |
| Number of times burned on arteries | 4.000 | .073 | -.833 | 10 | .424 | -.14286 | .17143 | -.52482 | .23911 |
| Number of times burned on liver | .009 | .924 | .061 | 10 | .953 | .71429 | 11.69532 | -25.34450 | 26.77307 |
| Number of times burned on gallbladder | .531 | .483 | 3.022 | 10 | **.013** | 17.65714 | 5.84267 | 4.63886 | 30.67543 |
| Percentage of connective tissue ablated | 3.135 | .107 | 1.275 | 10 | .231 | 2.33577 | 1.83215 | -1.74652 | 6.41806 |
| Percentage of adipose tissue ablated | 5.119 | .047 | -.079 | 10 | .939 | -.15535 | 1.96412 | -4.53169 | 4.22098 |
| Percentage of connective tissue clipped | 6.100 | .033 | .933 | 10 | .373 | .95516 | 1.02364 | -1.32566 | 3.23598 |
| Percentage of adipose tissue clipped | 1.563 | .240 | 4.336 | 10 | **.001** | 7.00946 | 1.61665 | 3.40734 | 10.61157 |

*Equal variances assumed*

Table S6: Automated LapSim performance metrics for junior vs senior trainees; difference mean results for laparoscopic appendicectomy by group, junior vs senior trainees

|  | Levene's Test for Equality of Variances | | t-test for Equality of Means | | | | | | |
| --- | --- | --- | --- | --- | --- | --- | --- | --- | --- |
|  | F | Sig. | t | df | Sig. (2-tailed) | Mean Difference | Std. Error Difference | 95% Confidence Interval of the Difference | |
|  |  |  |  |  |  |  |  | Lower | Upper |
| Total time | 6.824 | .024 | 2.843 | 11 | **.016** | 152.72931 | 53.71873 | 34.49519 | 270.96343 |
| Left instrument path length | 1.110 | .315 | 1.186 | 11 | .261 | 1.38997 | 1.17221 | -1.19004 | 3.96999 |
| Left instrument angular path | 1.004 | .338 | .506 | 11 | .623 | 162.17713 | 320.39177 | -543.00041 | 867.35467 |
| Right instrument path length | .822 | .384 | .978 | 11 | .349 | 1.88256 | 1.92586 | -2.35623 | 6.12134 |
| Right instrument angular path | 1.181 | .300 | 1.547 | 11 | .150 | 681.83983 | 440.68782 | -288.10752 | 1651.78717 |
| Left instrument outside view (n) | .535 | .480 | -2.184 | 11 | .051 | -6.78571 | 3.10637 | -13.62278 | .05135 |
| Left instrument outside view (s) | .159 | .698 | -3.210 | 11 | **.008** | -182.74900 | 56.92321 | -308.03613 | -57.46186 |
| Right instrument outside view (n) | .889 | .366 | .012 | 11 | .991 | .07143 | 6.04166 | -13.22618 | 13.36904 |
| Right instrument outside view (n | .118 | .737 | .437 | 11 | .671 | 3.96295 | 9.07572 | -16.01257 | 23.93848 |
| Blood loss | 2.991 | .112 | .840 | 11 | .419 | 37.69252 | 44.89609 | -61.12311 | 136.50815 |
| Energy applied in air | .179 | .680 | .012 | 11 | .991 | .09691 | 7.99435 | -17.49854 | 17.69236 |
| Total time burned on artery | 3.330 | .095 | -.566 | 11 | .583 | -1.62376 | 2.86820 | -7.93662 | 4.68910 |
| Number of times burned on artery | .884 | .367 | -3.174 | 11 | **.009** | -8.95238 | 2.82073 | -15.16076 | -2.74400 |
| Percentage of adipose tissue ablated | .040 | .846 | -.031 | 11 | .976 | -.18229 | 5.80371 | -12.95616 | 12.59158 |
| % of mesentery ablated | .238 | .635 | .275 | 11 | .788 | .37403 | 1.35950 | -2.61821 | 3.36626 |
| Percentage of adipose tissue ripped | .509 | .491 | -.688 | 11 | .506 | -1.09747 | 1.59467 | -4.60731 | 2.41237 |
| Percentage of mesentery ripped | 12.942 | .004 | 2.769 | 11 | **.018** | .38646 | .13955 | .07931 | .69361 |
| Appendix evacuated from body | .106 | .751 | -.238 | 11 | .817 | -.07143 | .30074 | -.73336 | .59050 |
| Incorrect stapler used | 4.874 | .049 | -.920 | 11 | .377 | -.14286 | .15530 | -.48467 | .19896 |

*Equal variances assumed*

Table S7: Automated LapSim performance metrics for junior vs senior trainees; difference mean results for laparoscopic right hemicolectomy by group, junior vs senior trainees

| **Independent Samples Test** | | | | | | | | | |
| --- | --- | --- | --- | --- | --- | --- | --- | --- | --- |
|  | **Levene's Test for Equality of Variances** | | **t-test for Equality of Means** | | | | | | |
|  | **F** | **Sig.** | **t** | **df** | **Sig. (2-tailed)** | **Mean Difference** | **Std. Error Difference** | **95% Confidence Interval of the Difference** | |
|  |  |  |  |  |  |  |  | **Lower** | **Upper** |
| Total time | .813 | .387 | -.807 | 11 | .437 | -123.58507 | 153.16246 | -460.69337 | 213.52322 |
| Left instrument path length | .002 | .966 | -.359 | 11 | .726 | -.38172 | 1.06284 | -2.72102 | 1.95757 |
| Left instrument angular path | .026 | .874 | -.207 | 11 | .840 | -48.78813 | 235.96718 | -568.14839 | 470.57212 |
| Left instrument outside view (n) | .030 | .867 | -.622 | 11 | .547 | -1.19048 | 1.91399 | -5.40315 | 3.02219 |
| Left instrument outside view (s) | 2.331 | .155 | -.857 | 11 | .410 | -29.38232 | 34.27610 | -104.82351 | 46.05887 |
| Right instrument path length | 8.860 | .013 | -.491 | 11 | .633 | -1.03952 | 2.11857 | -5.70246 | 3.62342 |
| Right instrument angular path | 4.180 | .066 | -.062 | 11 | .952 | -23.69296 | 384.85679 | -870.75705 | 823.37113 |
| Right instrument outside view (n) | .045 | .836 | 1.025 | 11 | .327 | 1.71429 | 1.67265 | -1.96720 | 5.39577 |
| Right instrument outside view (s) | 1.141 | .308 | -.087 | 11 | .932 | -.51386 | 5.92887 | -13.56321 | 12.53549 |
| Number of vessels injured | 1.496 | .247 | .189 | 11 | .854 | .09524 | .50457 | -1.01530 | 1.20578 |
| Correct vessels divided | 5.726 | .036 | -1.525 | 11 | .155 | -.52381 | .34339 | -1.27960 | .23198 |
| Burn time on artery | 9.031 | .012 | 1.938 | 11 | .079 | 4.00472 | 2.06645 | -.54351 | 8.55295 |
| Burn time on vein | 3.302 | .097 | 1.067 | 11 | .309 | 1.10663 | 1.03725 | -1.17634 | 3.38960 |
| Number of burns on artery | 4.026 | .070 | -.765 | 11 | .460 | -10.90476 | 14.24854 | -42.26558 | 20.45606 |
| Number of burns on vein | 4.307 | .062 | -.849 | 11 | .414 | -12.09524 | 14.25051 | -43.46040 | 19.26993 |
| Percentage of vein ablated | 4.874 | .049 | -.920 | 11 | .377 | -.30857 | .33545 | -1.04690 | .42975 |
| Total time artery overstretched | 24.275 | .000 | 2.031 | 11 | .067 | 2.94610 | 1.45046 | -.24634 | 6.13853 |
| Total time vein overstretched | .569 | .466 | .431 | 11 | .675 | .03626 | .08413 | -.14891 | .22143 |
| Number of times artery overstretched | 1.321 | .275 | 1.025 | 11 | .327 | .88095 | .85963 | -1.01109 | 2.77299 |
| Number of times vein overstretched | 2.643 | .132 | -.732 | 11 | .480 | -.92857 | 1.26883 | -3.72124 | 1.86410 |
| Vessels ripped | .114 | .742 | .171 | 11 | .867 | .04762 | .27878 | -.56596 | .66120 |
| Number of pressure damages on duodenum | 63.649 | .000 | -2.110 | 11 | .059 | -3.40476 | 1.61374 | -6.95658 | .14705 |

*Equal variances assumed*
